# Gadofosveset-Based Biomarker of Tissue Albumin Concentration: Technical Validation in Vitro and Feasibility in Vivo

**DOI:** 10.1002/mrm.25128

**Published:** 2014-02-11

**Authors:** Owen C Richardson, Octavia Bane, Marietta LJ Scott, Steven F Tanner, John C Waterton, Steven P Sourbron, Timothy J Carroll, David L Buckley

**Affiliations:** 1 Division of Medical Physics, University of LeedsLeeds, United Kingdom; 2 Departments of Biomedical Engineering and Radiology, Northwestern UniversityChicago, Illinois, USA; 3 Personalized Healthcare and Biomarkers, AstraZenecaMacclesfield, Cheshire, United Kingdom; 4 Department of Medical Physics and Engineering, Leeds Teaching Hospitals NHS TrustLeeds, United Kingdom.

**Keywords:** gadofosveset, albumin, biomarker, binding, relaxivity

## Abstract

**Purpose:**

There is currently no adequate method of mapping physiologic and pathophysiologic tissue albumin concentrations in human subjects. The objective of this study was to devise and evaluate a biomarker of regional albumin concentration using gadofosveset-enhanced MRI.

**Theory and Methods:**

A binding and relaxation model was devised and evaluated in vitro in solutions of albumin at 3.0 Tesla (T) and 4.7T. The method was evaluated in the heart in seven volunteers at 3.0T.

**Results:**

MRI-derived estimates of albumin concentration were in good agreement with true values over the range 0.1–1.0 mM (Pearson correlation coefficients of 0.85 and 0.88 for 3.0T and 4.7T, respectively). The mean calculated albumin concentration in the myocardium for the volunteers was 0.02 mM (range, 0.01–0.03 mM).

**Conclusion:**

Accurate estimates of albumin concentration in vitro suggest this may be a viable noninvasive alternative to existing techniques. In the myocardium the MRI-derived estimates of albumin concentration indicate the practical feasibility of the technique but were below expected values. Gadofosveset-enhanced MR relaxometry has potential in providing biomarkers of regional albumin concentration; further evaluation is required before it can be used reliably in vivo. Magn Reson Med 73:244–253, 2015. © 2014 Wiley Periodicals, Inc.

## INTRODUCTION

Albumin is the most abundant protein in human plasma, accounting for half of all serum proteins (1). It transports, by means of its numerous binding sites, endogenous compounds (2) and drugs (3), and is essential in regulating the flow of water between blood and tissue (1). Around 33% of albumin in the body is intravascular, with 49% in exchangeable extravascular locations and the remainder in remote extravascular compartments such as the skin (4). An imbalance in intra–extravascular albumin may potentially result in edema. Albumin concentrations may be accurately measured in urine or blood samples, with altered levels caused by changes in rates of synthesis, catabolism or extravascular leakage. Low levels of albumin have been linked to critical illness (5) and may be a risk factor for myocardial infarction (6). The body’s natural transcapillary exchange rate of around 5% of intravascular albumin per hour (7) may increase in damaged or angiogenic vessels. Localized increases in extravascular macromolecular content may be symptomatic of, for example, reperfused myocardial infarction (8) or tumor angiogenesis (9).

Although albumin concentrations in blood and urine are valuable indicators of albumin imbalance, they do not fully describe its biodistribution. Direct measurement of interstitial albumin concentration is not straightforward, with varying results found using invasive techniques such as wick implantation (10), blister suction (11) or double lumen catheterization (12). It is suggested that a noninvasive biomarker (13) of localized extravascular albumin may facilitate quantitative assessment of extravascular leakage. This may have prognostic and/or diagnostic value in assessment of tumor angiogenesis or myocardial infarction, for example, and may also be used for prospective assessment of response to treatment. Although conventional small-molecule gadolinium (Gd) contrast agents are frequently used in MRI to assess microvascular permeability, macromolecular Gd agents have shown increased sensitivity to malignancy (14), response to anti-angiogenic treatment (15) and ischemic microvascular damage (16).

Gadofosveset trisodium (Ablavar, Lantheus Medical Imaging, N Billerica, MA, previously marketed as Vasovist, Schering AG, Germany) is a Gd-containing contrast agent, with a stable gadopentetate core and phosphodiester linkage to a lipophilic albumin-binding group (17). In humans, over 90% of injected gadofosveset is reported to bind reversibly to serum albumin (18), increasing the effective molecular weight of the contrast agent from 957 Da to 68 kDa (19). Binding alters the pharmacokinetics of the molecule, reducing its extravasation and excretion rates; consequently the agent is well suited to angiography (20). Beyond angiography, recent studies have utilized gadofosveset in assessment of human brain tumors (21), liver lesions (22), chronic myocardial infarction (23), atherosclerosis (24), and liver fibrosis (25), and in combination with spin locking in vitro (26).

Substantially higher longitudinal and transverse relaxivities are observed for gadofosveset at low magnetic field strengths upon binding (27), due to the lower tumbling rate and longer correlation time of the bound molecule (28). The longitudinal and transverse relaxivities of the free (unbound) molecule are slightly higher than those of a conventional (nonbinding) small-molecule Gd-based agent such as gadopentetate (29). At physiologically applicable concentrations, it may be assumed that one gadofosveset molecule binds to a single albumin molecule (18,30). In this case, the bound fraction of gadofosveset is at a maximum where albumin exceeds gadofosveset concentration and declines where gadofosveset exceeds albumin concentration. This relationship suggests that, under certain conditions, it may be possible to use gadofosveset binding fraction as a biomarker for albumin concentration. However, signal intensity changes induced by the bound and free gadofosveset molecules cannot be directly separated in vivo and therefore binding fraction must be acquired through mathematical modeling.

This study aims to assess the viability of utilizing measured gadofosveset-enhanced longitudinal (R_1_, 1/T_1_) and transverse (R_2_, 1/T_2_) relaxation rates to develop a biomarker of albumin concentration in vitro. This method could be applied to generate a spatially located measure of tissue albumin which could be used as an alternative to current invasive techniques. Model feasibility is assessed using R_1_ and R_2_ measurements in vitro and in left ventricular blood and myocardial tissue of healthy human volunteers at 3.0 Tesla (T). Identification of abnormal extravascular albumin distribution correlating to increased capillary leakage may have several applications, including early indication of disease progression or treatment response in tumor angiogenesis, or assessment of reperfused myocardial infarction.

## THEORY

### Measuring Albumin Binding Fraction

For conventional Gd-based contrast agents, a single longitudinal relaxivity (r_1_) is usually sufficient to describe the relationship between the contrast-agent induced change in R_1_ (ΔR_1_) and gadofosveset concentration (C_g_); likewise a single transverse relaxivity (r_2_) describes the relationship between ΔR_2_ and C_g_:
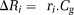
[1]where i = 1,2.

For albumin-binding gadofosveset, composite relaxivities are observed, comprising contributions from both the bound and free molecule. For the B_0_ field strengths used in this study, relaxivity of the bound molecule exceeds that of the free molecule. For the free molecule, the relationship in Eq. [1] may be assumed. For the bound molecule, there is a nonlinear relationship between C_g_ and R_1,2_ (although at high field r_1bound_ approaches r_1free_ and the relationship between C_g_ and R_1_ tends to linearity). The overall gadofosveset and serum albumin (C_sa_) concentrations may be defined as the sum of their bound and free components:
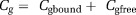
[2]
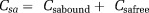
[3]

At low B_0_ field strengths, the distinct relaxivities of the bound and free gadofosveset molecules must be considered:
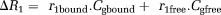
[4]
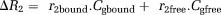
[5]

Assuming ΔR_1_ and ΔR_2_ can be measured and r_1bound_, r_1free_, r_2bound_, and r_2free_ are known, it is possible to rearrange Eqs. [4] and [5] to give expressions for bound and free gadofosveset concentration:
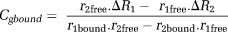
[6]
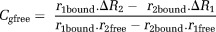
[7]

Combining Eqs. [6] and [7] according to Eq. [2] gives:
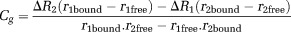
[8]

Bound, free, and overall gadofosveset concentrations can therefore be derived from measurement of ΔR_1_ and ΔR_2_.

### Measuring Albumin Concentration

In a second step, gadofosveset concentration is related to albumin concentration by assuming a chemical equilibrium between free and bound substances. The noncovalent binding equilibrium between a paramagnetic substrate and a protein is defined as (31):
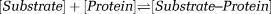


The association constant, or binding affinity (K_a_), involving a single equivalent binding site may be expressed as (31):
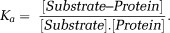


Expressing this binding affinity in terms of gadofosveset and albumin concentrations:
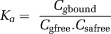
[11]

Assuming a single bound gadofosveset molecule per serum albumin molecule:
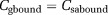
[12]

Eq. [9] becomes:
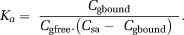
[13]

Rearranging for C_sa_:
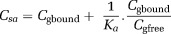
[14]

Inserting Eqs. [6] and [7] into Eq. [12] gives:
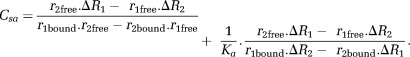
[15]

Eq. [13], therefore, provides a method for deriving albumin concentration through measurement of ΔR_1_ and ΔR_2_, assuming fixed relaxivity and binding affinity values.

### Measuring Bound Relaxivity

It remains to derive a method for measuring the relaxivity values from in vitro samples with known gadofosveset concentrations. Free relaxivity is derived using Eq. [1], applied to a solution without albumin. To derive a formula for bound relaxivity, C_gfree_ is first eliminated from Eq. [11] using Eq. [2], and the quadratic equation solved for C_gbound_. Inserting the result into Eq. [4] or Eq. [5] gives an expression for ΔR_1_ or ΔR_2_ where only bound relaxivity is unknown:
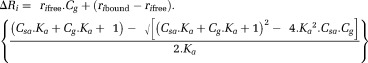
[16]where i = 1,2.

**Figure 1:**
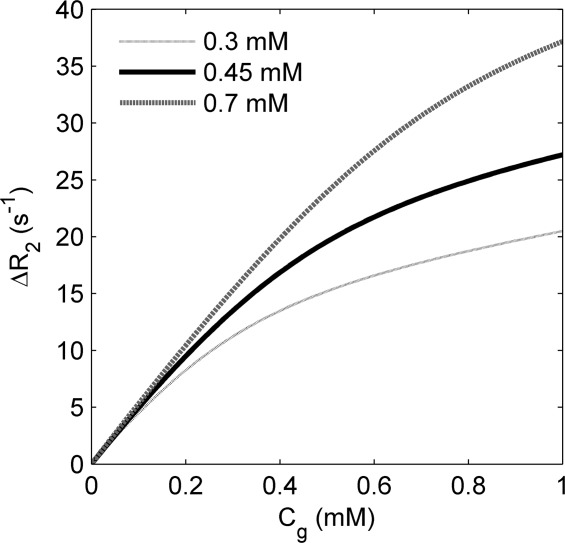
Modeled variation of ΔR_2_ with gadofosveset concentration for three serum albumin concentrations, based on Eq. [14], assuming values of K_a_ = 11.0 mM^−1^, r_2bound_ = 60.0 s^−1^ mM^−1^, r_2free_ = 6.1 s^−1^ mM^−1^ (29)

Only the negative form of the quadratic solution is applicable as the positive form would give a nonzero solution for C_gbound_ at C_g_ = 0. Eq. [14] has been represented in a similar form in several papers (for example, (30,32,33)). The model describes a gradual transition of binding fraction, from a maximum at low C_g_, where observed relaxivity is dominated by r_1,2bound_, toward a minimum at high C_g_, where r_1,2free_ has the greater influence (Fig. 1). As the model assumes a single binding site, the shift in emphasis from r_1,2bound_ to r_1,2free_ occurs at around C_g_ = C_sa_.

Accepting that r_2bound_ > r_2free_, it follows from Eq. [2] and Eq. [5] that, in all cases:
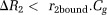
[17]

It should be noted that experimental imprecision in R_2_ measurement (and R_1_ measurement, as C_g_ is calculated using Eq. [8]) may violate this inequality, and may lead to calculated values of C_sa_ ≤ 0 mM. For transverse relaxivity, r_2bound_ is much higher than r_2free_ at all B_0_ values; for longitudinal relaxivity, r_1bound_ is much higher than r1_free_ at low B_0_ but both are effectively equivalent at very high B_0_ (34). This variation with field strength means that at low B_0_ any imprecision in R_2_ measurement has a much greater influence on calculated C_g_ (Eq. [8]), therefore, it is expected that the model may not be applicable at low B_0_ values.

## METHODS

### In Vitro Validation

Model validation was carried out by calculating C_sa_ (using Eq. [13]) for a range of in vitro solutions. This requires values of K_a_, ΔR_1_, ΔR_2_, r_2bound_, r_2free_, r_1bound_, and r_1free_. ΔR_1_ and ΔR_2_ were measured within the study, a fixed K_a_ value of 11.0 mM^−1^ was assumed in calculations (27,28,32), and relaxivity values were derived from the data presented in this study (as values for matching experimental conditions could not be found in the literature).

In vitro solutions of gadofosveset (Vasovist) were prepared for use at 3.0T with phosphate-buffered saline (PBS, dry powder reconstituted with deionized water, pH 7.4, Sigma Aldrich, St Louis, MO) and human serum albumin (HSA, Cohn fraction V lyophilized powder, Sigma Aldrich, in PBS). Solutions were created at C_sa_ and C_g_ concentrations between 0 and 1.0 mM; a total of 26 combinations of gadofosveset and HSA were prepared. A set of solutions containing the nonbinding contrast agent gadopentetate dimeglumine (Magnevist, Bayer Healthcare Pharmaceuticals, Germany) in HSA at C_sa_ = 0.7 mM were created to act as a control. The solutions measured at 4.7T used bovine serum albumin (BSA, Cohn fraction V lyophilized powder, Sigma Aldrich, in PBS) in place of HSA, within the same range of concentrations.

r_1free_ and r_2free_ were calculated by applying the linear model in Eq. [1] to the ΔR_1_ and ΔR_2_ values for the gadofosveset–PBS samples (C_sa_ = 0 mM), where no binding was assumed. To prevent the relaxation rates for any given gadofosveset–albumin sample(C_sa_ > 0 mM) influencing the relaxivity values subsequently used to calculate C_sa_ for that sample, bound relaxivity was calculated by setting aside one sample and applying a one-parameter model fit to the remaining subset of ΔR_1_ and ΔR_2_ values (using Eq. [14]). This process was repeated for each sample until a set of individual r_1bound_ and r_2bound_ values was created. The calculated relaxivities associated with each excluded sample (and its measured ΔR_1_ and ΔR_2_ values) were used in the subsequent C_sa_ calculation for that sample using Eq. [13].

### In Vitro Data Acquisition: 3.0T

Tubes were placed vertically within a cardiac coil in a 3.0T Philips Achieva TX system. Solutions were maintained at a temperature of 34–37°C with warm air flow, verified with a fiber optic temperature probe in an adjacent water tube. T_1_ values were measured using a spin echo inversion recovery sequence with 5 inversion times (TI = 50, 225, 371, 1665, 4875 ms), repetition time (TR) = 5000 ms, echo time (TE) = 6.2 ms. T_2_ values were measured using a multi-echo sequence with eight echo times (TE = 50, 100, 150, 200, 250, 300, 350, 400 ms), TR = 1000 ms. Additional parameters common to both T_1_ and T_2_ measurement: field of view = 231 × 231 mm; matrix size = 240 × 240 pixels; single coronal (horizontal) slice; slice thickness = 10 mm.

### In Vitro Data Acquisition: 4.7T

Tubes were placed vertically in a cylindrical cradle of diameter 60 mm and inserted into a 63-mm quad coil in a horizontal bore 4.7T magnet with Bruker console running ParaVision 5.1 software (Bruker BioSpin MRI GmbH, Ettlingen, Germany). Solutions were maintained at a temperature of 37°C with warm air flow, verified with a fiber optic temperature probe in an adjacent water tube. R_1_ values were measured using a RARE saturation recovery imaging sequence (35), with nine recovery times (57.2, 68.5, 78.5, 88.5, 103.5, 183.5, 283.5, 383.5, 983.5 ms) and a TE of 11 ms. R_2_ values were measured using a multi-slice multi-echo (MSME) sequence, with 20 equally spaced TE values from 11 to 220 ms and a TR of 1000 ms. Additional parameters common to both T_1_ and T_2_ measurement: field of view = 60 × 60 mm; matrix size = 256 × 256 pixels; RARE factor = 2; averages = 1; centric encoding; single coronal (horizontal) slice; slice thickness = 1 mm.

### Relaxation Rates

A circular region of interest (ROI) was drawn within each tube and the mean signal intensity (SI) of each ROI measured using ImageJ software (v1.42q, Rasband, W.S., ImageJ, U. S. National Institutes of Health, Bethesda, MD, http://imagej.nih.gov/ij/, 1997–2011). SI values at 4.7T were adjusted for noise bias using a simple Rician correction (36), based on mean standard deviations of four background regions in each image. R_1_ values at 4.7T and R_2_ values at 3.0T and 4.7T, along with 95% confidence intervals, were determined from two-parameter nonlinear fits to Eqs. [16] and [17], respectively, using MATLAB (v 7.9, MathWorks, Natick, MA). R_1_ calculation at 3.0T included an extra term for TR (Eq. 18).
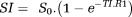
[18]
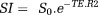
[19]
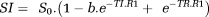
[20]where *S_0_* represents the fully recovered SI value and *b* is a factor accounting for imprecision in the 180° inversion pulse, applied to each ROI.

Contrast agent-induced changes in relaxation rate (ΔR_1,2_) were calculated by subtracting R_1,2_ values for each non-Gd C_sa_ solution (C_g_ = 0) from equivalent Gd-containing C_sa_ solutions (C_g_ > 0).

### In Vivo Feasibility Assessment: 3.0T

A total of seven healthy volunteers (five male, mean age 36 ± 10 years, mean weight 81 ± 15 kg) underwent pre- and postcontrast short-axis cardiac scans on a 3.0T Siemens Skyra system at Northwestern Memorial Hospital, Chicago. The study was approved by the Institutional Review Board (IRB) at Northwestern University, with informed consent obtained from all participants. IRB approval did not include provision for taking blood samples, therefore per-volunteer measures of hematocrit and blood albumin were not available.

Images were acquired as part of a larger study mapping flow patterns in thoracic aortic aneurisms (TAA) in different progression stages. Myocardial T_1_ and T_2_ values with administration of an MR contrast agent were also acquired to study changes of these parameters associated with inflammatory and connective tissue diseases that are in turn associated with the progression of TAA. A small timing bolus of 1.0–2.0 ml of gadofosveset (Ablavar) was used to establish arrival time and was followed by a main bolus of 6.2–8.8 mL, giving a total dose of 0.12 mL/kg (0.03 mmol/kg). A modified Look-Locker inversion recovery (MOLLI) sequence (37) with motion correction (38) (field of view = 270 × 360 mm, matrix size = 144 × 256 pixels, flip angle = 35°, TR = 313.45 ms, TE = 1.13 ms, bandwidth/pixel = 975 Hz) was used for T_1_, with T_1_ maps created inline by the system software. This version of the MOLLI sequence consisted of two inversions, with three images acquired after the first inversion (initial effective TI of 120 ms, and RR interval added to the other two acquisitions), and five images acquired after the second inversion (first effective TI of 200 ms; 200 ms + RR for subsequent acquisitions). Images were acquired with a specific trigger delay to select for end diastole. MOLLI acquisition was followed by a T_2_ mapping sequence using a single-shot T_2_-prepared steady-state free precession (SSFP) acquisition with three T_2_-preparation echo times: 0, 24, and 55 ms (39) (field of view = 337 × 450 mm, matrix size = 144 × 192 pixels, TR = 201.88 ms, TE = 1.07 ms, flip angle = 40°, bandwidth/pixel = 930 Hz). For all sequences, 8 mm slices were acquired at cardiac short axis base, mid and apex locations. Postcontrast images were acquired at up to three time points for each volunteer, with T_2_ image acquisition occurring 1–2 min after T_1_ acquisition (Table 1). The mid-point between T_1_ and T_2_ image acquisitions was used as the postcontrast reference time for each data point when plotting the results.

**Table 1:** Main Bolus and Image Acquisition Times for Volunteers^a^

Volunteer no.	Main bolus	Measurement					
		T_1_ [1]	T_2_ [1]	T_1_ [2]	T_2_ [2]	T_1_ [3]	T_2_ [3]
1	07:08	14:31	16:47	–	–	–	–
2	03:52	08:57	11:19	38:34	39:35	54:06	55:53
3	02:35	07:10	08:10	29:12	30:15	–	–
4	04:15	29:17	31:44	–	–	–	–
5	08:24	41:43	43:25	–	–	–	–
6	03:24	23:45	25:02	28:09	29:29	39:23	40:38
7	03:06	05:34	07:21	18:34	20:16	27:02	28:36

a Time from first administration of contrast agent (timing bolus), MM:SS.

ROIs were drawn within the left ventricle and within the myocardium on each pre- and postcontrast T_1_ and T_2_ map at the middle of the short axis view, and median and standard deviation values derived using MATLAB. For albumin calculation, each ROI is considered as a single well-mixed compartment, which is a valid assumption for the left ventricle, where gadofosveset is entirely intravascular, but is a simplification of conditions in the myocardium, where ΔR_1_ and ΔR_2_ are influenced by gadofosveset in vascular and extravascular spaces.

As the described T_2_ acquisition protocol is optimized for myocardial T_2_ measurement it is likely to underestimate the longer T_2_ of native blood, which may lead to an underestimation in ΔR_2_ calculation. To investigate this influence, an underestimation in ΔR_2_ was simulated and the effect on calculated albumin values observed. Expected ΔR_2_ values were obtained using Eq. [14] across a range of C_sa_ and C_g_ values, based on derived in vitro 3.0T relaxivities; these ΔR_2_ values were then reduced by an arbitrary 10% and C_sa_ values calculated according to Eq. [13].

## RESULTS

### In Vitro Data at 3.0T and 4.7T

Mean individual gadofosveset relaxivity values measured at 3.0T and 4.7T for the range of C_sa_–C_g_ combinations are given in Table2; standard deviations indicate the variance in calculated relaxivity.

**Table 2:** Mean Individual Gadofosveset Relaxivity Values (and SDs)

	3.0T		4.7T	
Relaxivity (s^−1^ mM^−1^)	This study (PBS/HSA)	Literature^a^	This study (PBS/BSA)	Literature^a^
r_1bound_	10.0 (0.1)	9.9	6.5 (0.0)	6.9
r_1free_	6.1 (0.3)	5.3	4.5 (0.1)	5.5
r_2bound_	100.9 (0.7)	60.0	60.0 (0.2)	60.0
r_2free_	7.5 (0.2)	6.1	10.7 (0.2)	6.9

a Literature values from (29), where r_1,2free_ are measured in water at 37°C and r_1,2bound_ are observed relaxivities in bovine plasma at 37°C.

**Figure 2:**
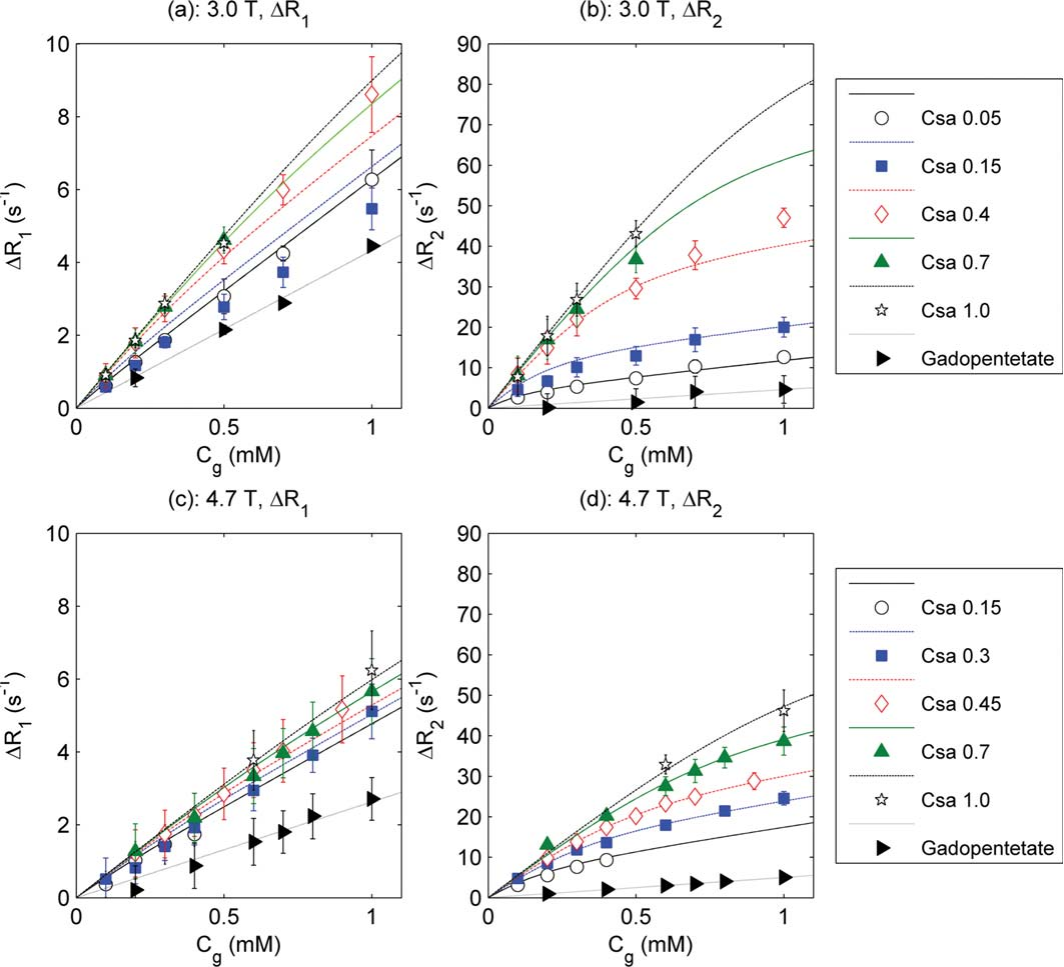
ΔR_1_ (left column) and ΔR_2_ (right column) values for gadofosveset at a range of albumin concentrations and for gadopentetate at 0.7 mM, at 3.0T (upper row) and 4.7T (lower row). Points represent measured values (with 95% confidence intervals); gadofosveset lines represent model fit based on relaxivities in Table 2; gadopentetate lines represent linear fit.

Figure 2 shows model fits (Eq. [14]) plotted against actual ΔR_1_ and ΔR_2_ gadofosveset data points at 3.0T and 4.7T, using the mean individual relaxivities in Table 2. A linear fit to the gadopentetate data is also shown.

**Figure 3:**
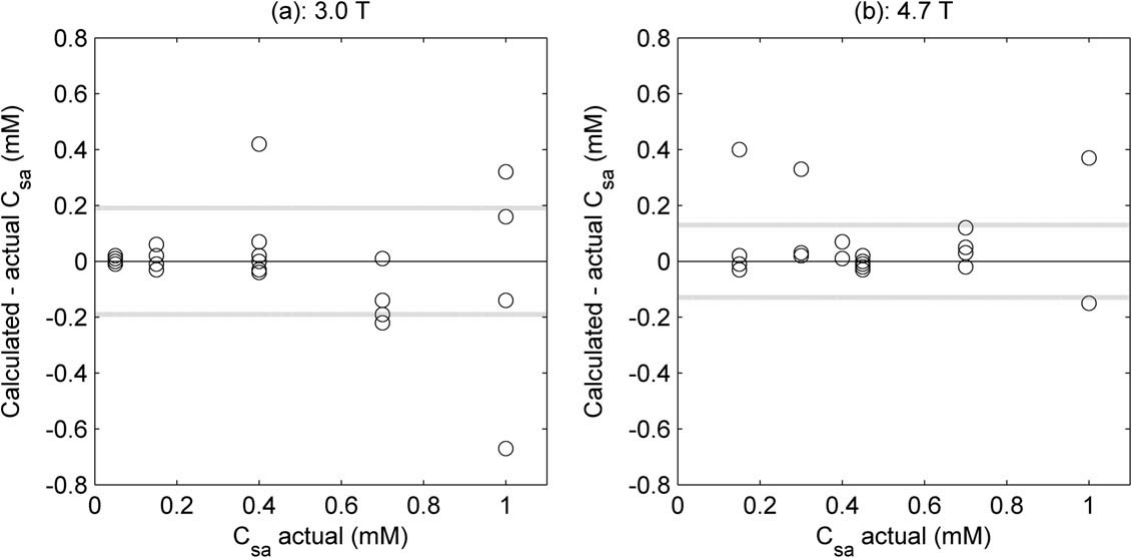
Bland–Altman plots of actual versus difference (calculated – actual) C_sa_ at 3.0T (26 plotted points) (a) and 4.7T (24 plotted points) (b). Dashed lines indicate standard deviation of difference

In Figure 3 calculated C_sa_ values (using Eq. [13]) are compared with actual values for each solution using individually derived relaxivity values at 3.0T and 4.7T. Four data points violated the inequality described in Eq. [15], and were therefore excluded from the 4.7T calculations. The model-derived C_sa_ values correlate with actual C_sa_ at a statistically significant level at both field strengths (Pearson correlation coefficients of 0.85 and 0.88 for 3.0T and 4.7T, respectively).

### Volunteer Data at 3.0T

**Figure 4:**
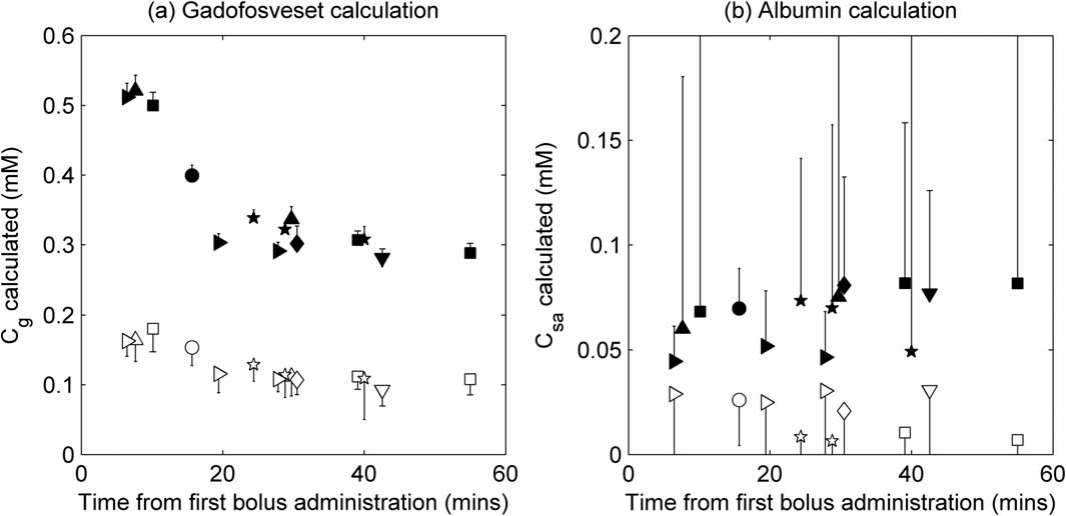
Calculated gadofosveset (a) and albumin (b) concentrations in myocardium (open symbols) and left ventricle (filled symbols) in healthy volunteers at 3.0T. Each symbol shape represents a different volunteer; values are plotted against time from first administration of contrast agent (to mid-point between T_1_ and T_2_ image acquisition times); error bars indicate uncertainty in calculations, calculated by propagation of errors using standard deviation of initial R_1_ and R_2_ ROI measurements (errors are symmetrical about data point, but only one side shown to aid clarity)

Precontrast T_1_ values in the left ventricle and myocardium were in the range 1493–1818 ms and 1099–1124 ms, respectively. Precontrast T_2_ values in the left ventricle and myocardium were in the range 117–158 ms and 43–47 ms, respectively. Calculated gadofosveset and albumin concentrations in the left ventricle and myocardium are shown in Figure 4, with data for all seven volunteers plotted against time from first bolus administration. The models for calculating gadofosveset (Eq. [8]) and albumin (Eq. [13]) concentrations used the 3.0T (PBS/HSA) relaxivity values shown in Table 2.

The effect of an underestimation in ΔR_2_ on calculated C_sa_ was simulated at a range of C_g_ values, using the 3.0T relaxivity values from Table 2. Simulations showed that a 10% underestimation in ΔR_2_ led to an underestimation in calculated C_sa_. This model underestimation increases as actual C_sa_ increases, and is more pronounced at lower C_g_. For example, at C_sa_ = 1.0 mM calculated C_sa_ is 0.6 mM lower than actual C_sa_ for C_g_ =0.1 mM, but calculated C_sa_ is only 0.2 mM lower than actual C_sa_ for C_g_ = 1.0 mM.

## DISCUSSION

Increased capillary leakage is symptomatic of a range of pathologies and healthy processes, resulting in rapid wash-in and wash-out of small molecule contrast agents and an increased transfer of macromolecules, including intravascular albumin, to the interstitial space. In vivo measurement of extravascular albumin content is not straightforward, although a range of invasive techniques are currently available. This study has explored the possibility of utilizing the albumin-binding properties of the Gd-based contrast agent gadofosveset to generate a novel and location-specific noninvasive method for measuring levels of albumin at moderate to high magnetic field strengths. Pre- and postcontrast R_1_ and R_2_ measurements are regularly carried out in MRI; the models presented here combine these changes in relaxation rate with calculated relaxivity values and a literature binding affinity value to produce a basic measure of tissue albumin concentration.

### In Vitro Model Validation

Calculated r_1_ and r_2_ relaxivity values at both 3.0T and 4.7T are in general agreement with previously published values (Table 2), although it is difficult to find directly equivalent experimental conditions for comparison. Using mean calculated relaxivity values, the model represents a good fit to gadofosveset ΔR_1_ and ΔR_2_ data points at low and high C_sa_ values (Fig. 2), suggesting that the assumption of a single binding site on the albumin molecule is adequate at these concentration levels. The primary binding site is known to provide the greatest contribution to relaxivity (28), and it is unlikely that C_g_ levels would be sufficiently high in vivo during the postbolus phase to necessitate inclusion of additional binding sites in this model (32). An attempt to model the data with second and third binding sites filled sequentially according to relative C_g_ and C_sa_ concentrations, using binding affinity values of 0.84 and 0.26 mM^−1^ (28), did not noticeably alter the model fits to measured data points (data not shown).

Excluding negative calculated C_sa_ values resulting from measurement imprecision and comparing the remaining calculated and actual C_sa_ values (Fig. 3), the model-derived C_sa_ values correlate with actual C_sa_ at a statistically significant level at both 3.0T and 4.7T.

The albumin-calculation model presented here is expected to work well at higher B_0_ field strengths (3.0T and above), where there is a large difference between r_2bound_ and r_2free_ but a small difference between r_1bound_ and r_1free_. At low fields, r_1bound_ is close to r_2bound_ and the difference between ΔR_1_ and ΔR_2_ is small. In this case, the precision of the model input parameters would be insufficient to overcome the sensitivity of the model to the variability in those parameters, leading to a breakdown of the model. At very high B_0_ field strengths, r_1bound_ and r_1free_ values for gadofosveset may be considered equivalent and the model may be simplified to incorporate a linear relationship between ΔR_1_ and C_g_. The C_g_ calculation described in Eq. [8] may then be represented as C_g_ = ΔR_1_/r_1_.

An underlying correlation between relaxivity and protein content has been shown in previous studies for Gd-based contrast agents not conventionally described as albumin binding (40,41). In vitro gadopentetate ΔR_2_ data points are well represented here by a linear fit (Fig. 2), suggesting no observable influence of weak binding on contrast agent relaxivity at the albumin levels used in this study. Without separate bound and free transverse relaxivities, gadopentetate provides no means of estimating C_sa_ through application of the model presented here. The high binding affinity of gadofosveset makes it a much more sensitive biomarker of albumin.

### In Vivo Feasibility

Gadofosveset-enhanced cardiovascular imaging is an area of active research (42–46), and likely to remain so in North America where the agent is available under the trade name Ablavar. One potential clinical application of the technique for calculating albumin concentration relates to myocardial infarction, therefore a feasibility assessment using human cardiac images was considered relevant. Cardiac imaging has the advantage of enabling direct comparison of calculated albumin values from blood in the left ventricle and from highly perfused myocardial tissue. However, before the model can be assessed, motion correction and other technical challenges must be overcome.

Precontrast T_1_ and T_2_ values generally correlate well with literature values (47–49), although longer T_2_ values in blood have been quoted elsewhere (50). Combining data from seven volunteers with images acquired at a range of time points gave remarkably consistent values of the two model input variables ΔR_1_ and ΔR_2_, and supported calculation of appropriate C_g_ values in both the left ventricle and the myocardium (Fig. 4a). As expected, gadofosveset concentration peaks at the earliest time points postbolus and decreases toward an equilibrium value, although this was not a dynamic acquisition therefore the temporal resolution is such that the bolus peak is not fully described.

At a dose of 0.03 mmol per kg, the average blood concentration of gadofosveset for an 81 kg adult with a total blood volume of 6.4 L would be 0.4 mM; allowing for some extravasation and excretion, the gadofosveset values calculated here in the left ventricle appear reasonable. For a small molecule agent such as gadopentetate, approximately 50% may diffuse to the extravascular space from the blood on the first pass through the capillary bed (51). Although, as a “blood pool” agent, gadofosveset may be expected to remain predominantly within the intravascular space, at high concentrations (immediately after bolus injection, for example) the bound fraction will be low and the extravasation rate may be similar to that of a conventional agent (52). A study in rabbits showed that 61% of injected gadofosveset was still in the blood at 1 min postinjection (32). Certainly, a reduction in C_g_ between the left ventricle and myocardium is expected, as noted in the relative values here.

Unlike gadofosveset, albumin concentration is expected to remain consistent within an individual for the image acquisition duration. Although there is some within-subject variability (Fig. 4b), this is a representation of the imprecision in data acquisition and does not correlate with time postbolus. The mean calculated albumin concentration in the left ventricle of the seven volunteers was 0.07 mM (range, 0.04–0.08 mM); in the myocardium the mean calculated C_sa_ was 0.02 mM (range, 0.01–0.03 mM).

A reference measure of albumin concentration was not available for comparison. Serum albumin levels in plasma (C_sa_plasma_) are expected to be approximately 3.5–5.0 g/dL (0.52–0.74 mM) (53). Assuming a hematocrit (Hct) of 0.42, this equates to albumin levels in whole blood of 0.30–0.43 mM (where blood concentration = C_sa_plasma_ (1 − Hct)). Previous studies quote interstitial fluid albumin concentrations (C_sa_interstitial_) of 0.2–0.4 mM (10,11,54). However, the myocardium ROI contains intravascular, extravascular extracellular and intracellular spaces. Neglecting the intracellular space, as gadofosveset cannot directly access it, and assuming an extracellular volume fraction (EVF) of 0.25 (55), a myocardial blood volume (MBV) of 8% (56) and a hematocrit in capillaries (Hct_cap_) of 0.25, tissue albumin (C_sa_tissue_, measurable using gadofosveset) may be expected to be in the range 0.07–0.11 mM (where C_sa_tissue_ = MBV. (C_sa_plasma_.(1 − Hct_cap_)) + C_sa_interstitial_. (EVF − MBV)). This range of expected values assumes that all blood vessels in the myocardium are capillaries; in reality, a proportion would be larger than capillaries and would, therefore, have a higher Hct, leading to a slightly lower range of expected C_sa_tissue_ values.

Calculated C_sa_ values were lower than might be expected in healthy volunteers. Factors contributing to an underestimation could include the relative timings of the T_1_ and T_2_ measurements, the separation of gadofosveset delivery into a timing bolus and a main bolus, and the potential incompatibility of translated in vitro relaxivities (for example, due to fundamental differences between in vitro and in vivo measurement of relaxivity (57), although the inclusion of albumin in the in vitro samples used for relaxivity calculation will moderate this issue). The selected scanning parameters were optimized for myocardial tissue and are likely to lead to an underestimation in ΔR_2_ in the left ventricle. The large error bars on the C_sa_ calculations in the left ventricle (Fig. 4b) confirm the difficulty in applying the model to left ventricular data acquired under conditions optimized for myocardial measurement. Simulated data suggest that underestimation in ΔR_2_ may result in a substantial underestimation in calculated C_sa_. However, it should be noted that the described method may not be appropriate or necessary in the left ventricle as albumin levels in blood can be readily measured from blood samples. The primary utility of the method may be in providing measurement of albumin concentration in tissue, where C_g_ is lower and C_sa_ is conventionally difficult to acquire.

A previous study using the contrast agent gadobenate (58), which has a much lower albumin binding affinity than gadofosveset (59), suggested that renal protein leakage could be identified by analyzing tubular flow differences following injection of two contrast agents, one binding and one nonbinding. Attempts have also been made to map protein levels by utilizing the distinct field dependency of the bound and free gadofosveset molecule (termed delta relaxation enhanced MR, DREMR) (60,61), although this approach requires the use of additional hardware to modulate B_0_ field strength. The advantage of the method described in this study over either of these approaches is that it only requires a single contrast agent injection and may be readily derived from routinely acquired R_1_ and R_2_ measurements using conventional equipment.

## CONCLUSIONS

The model presented here demonstrates the feasibility of determining in vitro serum albumin concentration using pre- and postgadofosveset measurements of R_1_ and R_2_ at high B_0_ fields. The method was successfully validated using in vitro samples at 3.0T and 4.7T. Feasibility assessment in a small number of human volunteers was performed using cardiac images, and consistent ΔR_1_, ΔR_2_ and C_g_ values were determined. Underestimation of C_sa_ may be the result of several contributing factors, including the timing of the image acquisitions and translation of in vitro relaxivities. A more effective application of this method may be in tumor angiogenesis, where increased extravascular macromolecular leakage rates are observed and imaging is less restricted by cardiac or respiratory motion. Further in vivo assessment is suggested, to include: simultaneous T_1_ and T_2_ measurement; additionally acquiring images between the timing bolus and the main bolus; and blood sampling to establish hematocrit and reference blood albumin levels.

This novel approach may enable noninvasive assessment of extravascular leakage of albumin, utilizing parameters acquired during routine imaging, in regions where implementation of invasive techniques for measurement of interstitial albumin is conventionally challenging. A range of potential clinical applications are envisaged, including assessment of myocardial infarction, tumor angiogenesis, and response to treatment.
